# No Obvious Role for Suspicious Oral Pathogens in Arthritis Development

**DOI:** 10.3390/ijerph18189560

**Published:** 2021-09-10

**Authors:** Poerwati S. Rahajoe, Menke J. de Smit, Elisabeth Raveling-Eelsing, Marines du Teil Espina, Tim Stobernack, Paola Lisotto, Hermie J. M. Harmsen, Jan Maarten van Dijl, Nyoman Kertia, Arjan Vissink, Johanna Westra

**Affiliations:** 1 Department of Oral and Maxillofacial Surgery, Dr. Sardjito General Hospital, Yogyakarta 55281, Indonesia; ibmfkgugm@yahoo.co.id; 2 University Medical Center Groningen, Department of Oral and Maxillofacial Surgery, University of Groningen, P.O. Box 30.001, 9700 RB Groningen, The Netherlands; a.vissink@umcg.nl; 3 University Medical Center Groningen, Department of Rheumatology and Clinical Immunology, University of Groningen, P.O. Box 30.001, 9700 RB Groningen, The Netherlands; e.raveling-eelsing@umcg.nl (E.R.-E.); johanna.westra@umcg.nl (J.W.); 4 Center for Dentistry and Oral Hygiene, University Medical Center Groningen, University of Groningen, Antonius Deusinglaan 1, 9713 AV Groningen, The Netherlands; 5 University Medical Center Groningen, Department of Medical Microbiology, University of Groningen, P.O. Box 30.001, 9700 RB Groningen, The Netherlands; m.du.teil.espina@umcg.nl (M.d.T.E.); t.stobernack01@umcg.nl (T.S.); p.lisotto@umcg.nl (P.L.); h.j.m.harmsen@umcg.nl (H.J.M.H.); j.m.van.dijl01@umcg.nl (J.M.v.D.); 6 Department of Rheumatology and Clinical Immunology, Dr. Sardjito General Hospital, Yogyakarta 55281, Indonesia; kertianyoman@yahoo.com

**Keywords:** rheumatoid arthritis, periodontitis, *Aggregatibacter actinomycetemcomitans*, *Porphyromonas gingivalis*, rheumatoid factor, anticitrullinated protein antibodies, risk factors

## Abstract

A particular role for *Porphyromonas gingivalis* (Pg) and *Aggregatibacter actinomycetemcomitans* (Aa) has been suggested in periodontitis and rheumatoid arthritis (RA), as these bacteria could initiate the formation of rheumatoid factor (RF) and anticitrullinated protein autoantibodies (ACPA). We assessed whether serum antibodies against Pg and Aa in RA patients and non-RA controls reflect the subgingival presence of Pg and Aa, and evaluated the relationship of these antibodies to the severity of periodontal inflammation and RA-specific serum autoantibodies. In 70 Indonesian RA patients and 70 non-RA controls, the subgingival presence of Pg and Aa was assessed by bacterial 16S rRNA gene sequencing, and serum IgG levels specific for Pg and Aa were determined. In parallel, serum levels of ACPA (ACPA:IgG,IgA) and RF (RF:IgM,IgA) were measured. The extent of periodontal inflammation was assessed by the periodontal inflamed surface area. In both RA patients and the controls, the presence of subgingival Pg and Aa was comparable, anti-Pg and anti-Aa antibody levels were associated with the subgingival presence of Pg and Aa, and anti-Pg did not correlate with ACPA or RF levels. The subgingival Pg and Aa were not related to RA. No noteworthy correlation was detected between the antibodies against Pg and Aa, and RA-specific autoantibodies.

## 1. Introduction

Periodontitis is epidemiologically associated with rheumatoid arthritis (RA), a chronic autoimmune disease characterized by inflammation of the joints, causing pain, bone damage, and disability, although the directionality of this relationship is not known [1]. The development of RA is a multistep process in which early phases are characterized by the presence of genetic and environmental risk factors and the systemic autoimmunity associated with RA, such as rheumatoid factor (RF) and anticitrullinated protein antibodies (ACPA) [2]. Periodontitis shares genetic and environmental risk factors with RA, and periodontitis by itself has been regarded as a risk factor for RA development [3,4].

RA was hypothesized to start at chronically inflamed mucosal surfaces, as encountered in periodontitis [5]. In addition to chronic mucosal inflammation, infection with specific periodontal pathogens has been implicated in the initiation of RA. Much attention has been focused on *Porphyromonas gingivalis* (Pg), which secretes a peptidyl arginine deiminase enzyme (PAD) that can citrullinate bacterial and human proteins [6,7,8]. Rosenstein et al. [3] suggested that PAD-induced antigens lead to the production of RF-containing immune complexes. In addition, this unique characteristic of Pg led to the hypothesis that the breakdown of tolerance against citrullinated proteins and the formation of ACPA are initiated by PAD-dependent citrullination [6,9]. Periodontal infection by *Aggregatibacter actinomycetemcomitans* (Aa) has also been implicated in the initiation of autoimmunity in RA. A secreted leukotoxin of Aa (LtxA) was shown to dysregulate activation of the human PAD enzymes in host neutrophils, leading to the release of hypercitrullinated proteins that mimic the repertoire of citrullinated antigens found in the RA joint [10].

A recent meta-analysis concluded that periodontitis may represent a risk factor for RA because of the genetic risks, bacterial infection, and proinflammatory profile shared between both diseases, although there was considerable heterogeneity among studies [11]. The reasons for conflicting epidemiological findings might be caused by the nonspecific classification criteria applied to periodontitis, the size of the patient cohorts used in the studies, and the lack of data regarding confounding factors and treatments [11]. It was suggested that, for a better understanding of the relationship between periodontitis and RA, retrospective studies without clinical periodontitis measurements could instead focus on the presence of Pg infection, which may be estimated objectively by measuring antibody levels [9]. Furthermore, it was suggested that anti-Pg antibodies might have predictive value for arthritis development [12]. The major objective of our study was to evaluate the extent to which serum antibodies against Pg and Aa in RA patients and non-RA controls (NC) reflect the subgingival presence of Pg and Aa, and to assess whether antibodies against these two pathogens can be related to the severity of periodontal inflammation and the levels of serum autoantibodies specific for RA. The results of this study reveal that, in our Indonesian study population, there was no obvious role of suspicious oral pathogens in arthritis development. Serum antibody levels against Pg and Aa indeed reflect subgingival infection with these oral pathogens; however, they cannot be used as a surrogate measure for periodontitis.

## 2. Materials and Methods

### 2.1. Patients

The included subjects were derived from a previously described Indonesian cohort [13]. In 70 RA patients and 70 NC with known periodontal status, the subgingival presence of Pg and Aa 16S rRNA was determined, as well as the serum antibodies to these periodontal pathogens (anti-Pg and anti-Aa). The inflammatory burden of periodontitis was assessed by the periodontal inflamed surface area (PISA) [14]. Two RA patients from the original cohort were excluded because of the absence of microbiological data. The non-RA controls were matched to the RA patients according to age, sex, smoking status, and number (Table 1). The factors that can influence the PISA are systemic disease, pregnancy, or the use of medication. Patients with diabetes, cardiovascular disease with anticoagulant medication, or that exhibited the presence of nonoral infection or malignancy, or patients that used antibiotics <3 months prior the study were excluded. Pregnancy, including a 6-month post-partum period, and breastfeeding were also exclusion criteria. Medication for hypertension was recorded. The 6 patients using calcium channel blockers all had a low percentage of sites with bleeding on probing (range 0–9.4%). Other medication for hypertension that can cause gingival overgrowth (such as nifedipine) was not used.

The STROBE guidelines for human observational studies were followed. In short, RA patients were consecutively recruited at the outpatient Rheumatology Clinic, Sardjito Hospital, University of Gadjah Mada, in Yogyakarta, Indonesia. The inclusion criteria were in conformity with the 2010 RA classification criteria of the American College of Rheumatology (ACR) [2]. Exclusion criteria were: under 18 years of age, edentulism, diabetes, cardiovascular disease with anticoagulant medication, the presence of nonoral infection, antibiotic use <3 months prior to the study, the presence of malignancy, and pregnancy including a 6-month post-partum period, as well as breastfeeding. Consecutive NC were selected from patients with an appointment for a first consultation for a third molar assessment or tooth extraction at the Oral and Maxillofacial Surgery Clinic, Sardjito Hospital, or the Dental Hospital, Gadjah Mada University in Yogyakarta, Indonesia. Inclusion criteria for the NC were: not suffering from RA and the absence of any distinct oral pathology other than periodontitis. The same exclusion criteria were applied as for the RA patients. The subjects were matched according to age, sex, and smoking status. This study was reviewed and approved by the Medical and Health Research Ethics Committee of the Medical Faculty of Gadjah Mada University, Yogyakarta, Indonesia, according to the Declaration of Helsinki 2008 (Ref: KE/FK/430/EC). All the patients were informed verbally and by letter, and the participants provided written informed consent.

All periodontal measures and subgingival samplings were taken by the same examiner (PSR) who was calibrated by a periodontist (MJdS) that was trained in performing PISA and subgingival sampling.

### 2.2. Subgingival Microbial Sampling

Subgingival microbial samples were taken from the deepest bleeding site per quadrant based on pocket probing depth measurements. If there were no bleeding periodontal pockets, the mesial site of the first molar, or in the absence of the first molar, the mesial site from the adjacent anterior tooth in the dental arch, was selected. Sample sites were isolated with cotton rolls, and supragingival plaque was carefully removed with curettes and cotton pellets. Subsequently, 2 sterile paper points per site (Inline, BM Dentale, Turin, Italy) were inserted and left in place for 10 s. Paper points were pooled per patient and stored in a sterile microtube (Stardec, TG Medical SDN PHD) at −20 °C.

### 2.3. DNA Extraction and 16S rRNA Gene Sequencing

Three paper points of the microbial sample were added to 0.5 g of 0.1 mm zirconia beads (BioSpec Products Inc., Bartlesville, OK, USA) in 500 µL sterile phosphate-buffered saline and were beaten at 5.5 m/s for 3 × 1 min at room temperature (Precellys 24, Bertin Technologies, Montigny-le-Bretonneux, France). Hereafter, samples were heated at 95 °C for 15 min and centrifuged at 4 °C. The supernatant was collected, 2 µL of 10 mg/mL RNase H (Promega, Madison, WI, USA) was added, and it was incubated for 15 min at 37 °C. Microbial DNA was isolated using the DNeasy Blood & Tissue Kit (Qiagen) according to the manufacturer’s instructions. The purity and concentration of the DNA were measured with a NanoDrop 1000 spectrophotometer (Thermo Scientific, Waltham, MA, USA). Illumina MiSeq preparation, and the sequencing and analysis of sequence reads were performed as described in Heida et al. [15]. For this study, the presence of Pg and Aa 16S rRNA was assessed. In short, the V3-V4 region of bacterial 16S rRNA genes was amplified by PCR with modified 341F and 806R primers containing a 6-nucleotide index sequence on the 806R primer [16]. FASTQ files of Illumina paired-end reads were processed with PANDAseq. The taxonomy of the phylum, family, and genus levels was determined using QIIME, and ARB was used to classify the bacteria at the species level [17,18,19]. Thereafter, the relative abundance of Pg and Aa was calculated by summing all counts of all species measured, and dividing the counts of each species with the sum of total counts, as percentages. For this study, we used the relative abundance for Pg an Aa only. A description of the entire microbiome is beyond the scope of this study.

### 2.4. Antibody Measurements in Serum

The IgG antibodies for Pg and Aa were measured by an in-house ELISA, in which pooled lysates of four randomly selected clinical isolates of Pg or Aa from patients with periodontitis were used as an antigen. Standard curves were made from protein standards (N protein-standard SL; Siemens Healthcare Diagnostics, Erlangen, Germany). The detection of anti-Pg and anti-Aa antibodies was carried out with mouse antihuman IgG (Fc fragment specific, clone JDC-10, Southern Biotech, Birmingham, AL 35209 USA). The variation coefficient was 20% [20].

The serum RA autoantibodies (ACPA and RF) were determined as described previously [13]. In short, IgG anticyclic citrullinated protein antibody (anti-CCP) levels were measured using a commercial anti-CCP2 kit (Euro Diagnostica, Arnhem, The Netherlands) according to the manufacturer’s protocol. Intra-assay variation was <10% and inter-assay variation was <12.5%. The IgA anti-CCP measurement was performed using a modification of the anti-CCP2 kit with goat antihuman IgA-HRP (Southern Biotech, Birmingham, Alabama 35209 USA), with a conjugate dilution of 1:4000 (10). The IgA seropositivity was defined as >2 SD above the mean of the never-smoking healthy controls without periodontitis in the present cohort, *n* = 50).

The RF IgM and IgA levels were determined using an in-house validated ELISA [21], and they are expressed in international units (IU) per milliliter. Inter- and intra-variation were <10%. Seropositivity was defined as >5 IU/mL for IgM RF, and >25 IU/mL for IgA RF.

### 2.5. Statistics

The major objective of our study was to evaluate to what extent serum antibodies against Pg and Aa in RA patients and the NC reflect the subgingival presence of Pg and Aa, and to assess whether antibodies against these two pathogens can be related to the severity of periodontal inflammation and the levels of serum autoantibodies specific for RA. Based on our own previous data [20,22], we calculated post hoc a statistical power of 98.8% in the difference of IgG anti-Pg antibody levels between a subgroup (*n* = 44) of non-RA individuals with (*n* = 19), and without (*n* = 25), cultivable subgingival Pg (Pg-negative mean 2.96 µg/mL, SD 3.82, and Pg-positive mean 66.38 µg/mL, SD 65.60, alpha 0.05; htpps://clincalc.com/stats/Power.aspx, accessed on 19 May 2021). Based on the literature, the prevalence of subgingival Pg is hardly different between RA and non-RA subjects and ranges from around 20% [20] to 80% [23]. Aiming for the inclusion of around 20 RA and 20 non-RA individuals positive for subgingival Pg, based on an estimated prevalence of 30% Pg positivity, we calculated that we required 67 individuals per group.

Statistical analyses were performed using GraphPad Prism software (version 8 for Windows; GraphPad Software). All data, except for age and DAS28, were non-normally distributed (based on QQ plots). For group comparisons, a Mann–Whitney U test was used for continuous variables, and a Fisher’s exact test was used for categorical variables. The correlations between different parameters were assessed by Spearman’s ρ. The significance level α was 0.05.

## 3. Results

### 3.1. Patients

The participant characteristics are listed in Table 1. Both cohorts were similar with regard to age, sex distribution, and smoking behavior. The median RA disease duration was 24 months, and 60% of the patient cohort presented high disease activity. The six patients using calcium channel blockers all had a low percentage of sites with bleeding on probing (range 0–9.4%).

A PISA value of ≥130 mm^2^ has been shown to be associated with the case definition of periodontitis as defined by the Center for Disease Control and Prevention and the American Academy of Periodontology (CDC-AAP) [24]. The prevalence of periodontitis according to this cut-off was not significantly different between RA patients and the NC (Table 2). In the RA patients, there was no difference in the PISA between patients treated with DMARDs and those that were not (*p* = 0.908).

Subgingival Pg was detected in around 80% of the RA patients and the NC, while Aa tended to be more frequently detected in the NC than in RA patients. For details on periodontal characteristics, see Table 2.

**Figure 1:**
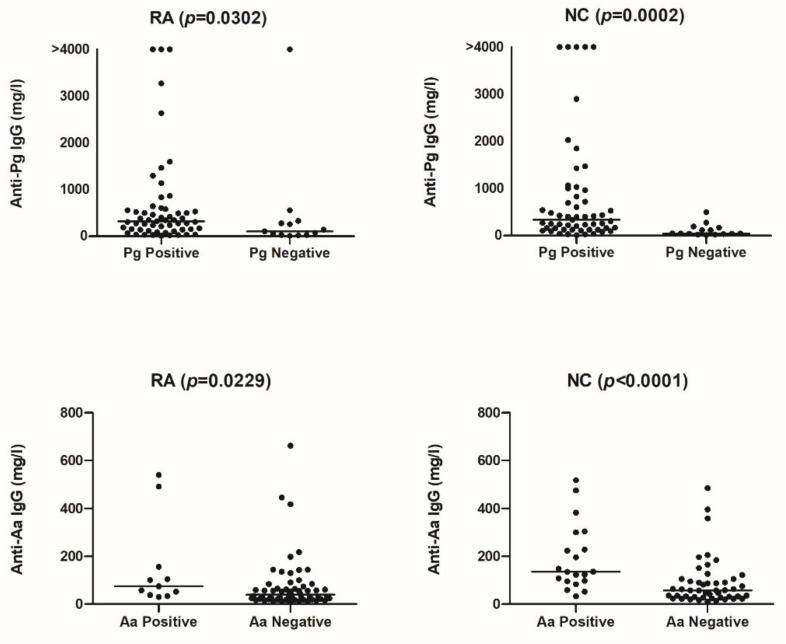
Levels of anti-*P. gingivalis* (Pg) and anti-*A. actinomycetemcomitans* (Aa) antibodies in RA patients and non-RA controls (NC) positive or negative for these bacteria. Lines represent the median.

**Figure 2:**
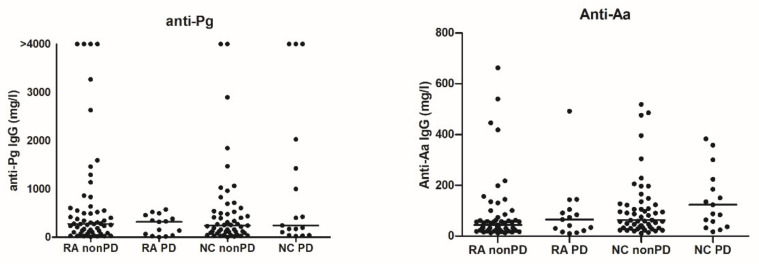
Levels of anti-*P. gingivalis* (Pg) and anti-*A. actinomycetemcomitans* (Aa) antibodies in relation to the presence or absence of periodontitis (PD) in RA patients and non-RA controls (NC). Lines represent the median.

Anti-Pg antibodies were detected in all sera, but the levels were significantly higher when subgingival Pg was identified. The same was observed for the levels of anti-Aa antibodies (Figure 1). No significant differences were observed between RA patients and the NC with regard to the levels of anti-*P. gingivalis* (Pg) and anti-*A. actinomycetemcomitans* (Aa) antibodies in relation to the presence or absence of periodontitis (PD) (Figure 2; anti-Pg: RA-non-PD: median 278 mg/L (IQR 106–606), RA-PD: 321 mg/L (41–459), NC-nonPD: 242 mg/L (102–534), NC-PD: 245 mg/L (74–1729). Anti-Aa: RA-non-PD: median 44 mg/L (IQR 24–74), RA-PD: 65 mg/L (21–105), NC-non-PD: 63 mg/L (33–125), NC-PD: 123 mg/L (47–262)).

RA patients with no medication or treated only with herbal medication (*n* = 8) had higher anti-Aa levels compared to RA patients with medication (*p* = 0.014). Moreover, the levels of anti-Pg tended to be higher in RA patients without medication compared to RA patients with medication (*p* = 0.066, data not shown).

### 3.2. Correlations

The detection of subgingival Pg and Aa was highly correlated with the levels of anti-Pg and anti-Aa in RA patients (ρ = 0.35, *p* < 0.003 for Pg, ρ = 052, *p* < 0.000 for Aa) and in the NC (ρ = 0.38, *p* = 0.001 for Pg, ρ = 0.29, *p* = 0.014 for Aa) (Table 3). The average relative abundance of Pg was 1.966 (SD 4.439) for RA, and 2.558 (SD 4.969) for NC. The average relative abundance for Aa was 0.0632 (SD 0.360) for RA, and 0.3368 (SD 1.632) for NC.

## 4. Discussion

In the present study, we showed that in both RA patients and the NC from an Indonesian cohort, the levels of serum anti-Pg and anti-Aa antibodies were associated with the detection of subgingival Pg and Aa. This implies that the measurement of antibodies against whole bacterial cell lysates of Pg or Aa is indicative of a subgingival Pg or Aa infection. As serum IgGs against Aa and Pg were previously shown to be extremely stable over 15 years in subjects with or without periodontitis; these levels indicate an infectious encounter with these pathogens at some point in time [25].

In our present cohort, the detection of subgingival Pg and Aa was comparable for RA patients and the NC, and the levels of anti-Pg and anti-Aa were comparable for RA patients and the NC at the group level. No noteworthy correlation was detected between antibodies against Pg and Aa- and RA-specific autoantibodies. In addition, antibodies against these two pathogens could not be related to the severity of periodontal inflammation. RA medication might influence anti-Pg and anti-Aa levels, although the group of RA patients without medication was small.

The prevalence of subgingival Pg in RA patients and the NC, and its possible relationships to antibodies or autoantibodies, has been investigated in several studies. However, no other group has investigated IgA antibodies, which are specific for mucosal immunity. In a previous study by our group, we observed that the subgingival prevalence of Pg was not different for the RA patients and the NC, and that there was no correlation detectable between the anti-Pg levels and arthritis autoantibodies in the serum [20]. Based on PCR analyses, Mikuls et al. [26] reported a 67% overall prevalence of subgingival Pg in RA patients, but this study did not address the possible correlations between subgingival Pg and ACPA and RF. Nevertheless, the authors reported a weak, but significant, correlation between the anti-Pg outer membrane antigen levels and ACPA (ρ = 0.14) and RF (ρ = 0.19) in RA patients (*n* = 287).

Schmickler et al. [27] showed by PCR that the prevalence of periodontal bacteria was not different for RA patients and the NC, but that higher numbers of *Fusobacterium nucleatum* and Pg were found in ACPA seropositive patients with RA. These different observations may relate to the use of different detection techniques and the inclusion of different patient groups. For instance, Scher et al. [23] analyzed the subgingival colonization of Pg by 16S rRNA pyrosequencing and identified Pg in 55% of patients with new-onset RA (without RA medication, *n* = 31), in 47% of patients with chronic RA (*n* = 34), and in 27% of healthy controls (*n* = 18). The difference in Pg prevalence could, however, be explained by the higher numbers of periodontitis cases in their RA population. When combining the findings from 16S rRNA pyrosequencing and/or a positive anti-Pg P18γ antibody response (against a chaperone peptide), the overall exposure to Pg was essentially the same for RA patients (78%) and the controls (83%), which is comparable to our present findings. Thus, it appears from both this study and ours that, overall, the exposure to Pg is relatively high in patients with and without (present) periodontitis.

The subgingival prevalence of Pg in a Norwegian cohort of 78 established RA patients was 22%, which is much lower than observed in the present study, although the same sequencing techniques were used [28]. Nonetheless, similar to our findings, no correlation of Pg with ACPA or RF in the serum of RA patients was detected. Furthermore, a recent meta-analysis showed that anti-Pg antibodies were positively correlated with RF and ACPA in RA patients, but the correlation coefficients were low (0.07 for RF, 0.15 for ACPA) [29]. Lastly, Konig et al. [10] investigated antibodies against the leukotoxin LtxA of Aa in RA patients and reported an association of anti-LtxA with ACPA and RF. However, it seems likely that the anti-LtxA levels are more representative for subgingival Aa infection [30], as there was no particular association with RA, with ACPA, or with the HLA SE alleles, which are the most important genetic risk factors for RA [10].

This study has some limitations. As radiographic assessment is necessary to confirm a periodontal diagnosis, the PISA in our study does not necessarily reflect the periodontal diagnosis, but the inflammatory burden. Therefore, the division of the presence or absence of periodontitis according to the cut-off is somewhat artificial. Furthermore, the prevalence of periodontitis according to the PISA was relatively low in our groups, but this is a common finding amongst women in Indonesia [31]. In the latter study, the burden of periodontitis was also comparable amongst RA patients and the matched NC. The vast majority of the cohort of this study was female (88%). The cohort was of Indonesian descent and, in this country, women usually do not smoke. An explanation for the lower values of ACPA might be the extremely low number of smoking RA patients, as smoking has been shown to be associated with an increased risk in ACPA-positive RA in the Malaysian population, which is geographically closely related to the Indonesian population [32]. Despite antirheumatic treatment, the majority of the RA patients had high RA disease activity. This might be due to the limited access to biological treatment for many patients in Indonesia because of the high costs [33]. Antirheumatic treatment seems to have a negligible influence on the periodontal condition of RA patients, and to not have an influence on the subgingival presence of Pg and Aa, accordingly [34,35].

## 5. Conclusions

The present study shows that, in our Indonesian cohort, antibody levels against whole bacterial cell lysates of Pg or Aa reflect subgingival infection by Pg or Aa, which was equally prevalent in RA patients and the non-RA controls. Importantly, no evidence for an association of antibodies against these periodontal pathogens and RA-specific autoantibodies was detected. Thus, it seems that the levels of antibodies against these two oral pathogens have no predictive value for arthritis development, as has been shown by our group for anti-Pg in clinically suspect arthralgia patients [22,36].

## Figures and Tables

**Table 1:** Characteristics of patients with rheumatoid arthritis (RA) and non-RA controls (NC) modified after [13].

Subject Characteristics	RA (*n* = 70)	NC (*n* = 70)	*p* Value
Age (mean, SD)	47.3 (11.1)	46.7 (11.9)	0.753
Female (*n*) (%)	62 (88.6)	62 (88.6)	1
Current smoking (*n*) (%)	2 (2.9)	1 (1.4)	1
Never smoking (*n*) (%)	7 (10.00)	6 (8.6)	1
**Co-morbidity (*n*) (%):**			
Asthma (+bronchodilator)	0 (0)	3 (4.3)	0.245
Hypertension (+medication)	7 (10.0)	4 (5.7)	0.532
Medication for hypertension, also combinations (*n*):			
Calcium channel blocker	4	2	
ACE inhibitor	2	2	
Angiotensin receptor blocker	2	0	
**RA patient characteristics:**			
Disease duration in months (median, IQR)	24 (6–48)		
DAS28 (mean, SD)	5.1 (1.1)		
Disease remission (DAS28 < 2.6) (*n*) (%)	0 (0)		
Low activity (DAS28 ≥ 2.6 < 3.2) (*n*) (%)	3 (4.3)		
Moderate activity (DAS28 ≥ 3.2 < 5.1) (*n*) (%)	25 (35.7)		
High activity (DAS28 ≥ 5.1) (*n*) (%)	42 (60.0)		
**Medication for RA (*n*) (%), also combinations:**			
None	8 (11.4)		
Herbal	5 (7.1)		
NSAID	44 (62.9)		
Steroid	55 (78.6)		
DMARD total	40 (57.1)		
DMARD specific (*n*):			
Methotrexate	32		
Leflunomide	2		
Chloroquine	2		
Sulfasalazine	11		
**ACPA seropositive (*n*) (%):**			
IgG (>10 U/mL)	29 (41.4)	0 (0)	<0.000
IgA ^†^ (>1.26 U/mL)	27 (38.6)	3 (4.3)	<0.000
**RF seropositive (*n*) (%):**			
IgM (>5 IU/mL)	34 (48.6)	1 (1.4)	<0.000
IgA (>25 IU/mL)	25 (35.7)	0 (0)	<0.000

RF: rheumatoid factor; Ig: immunoglobulin; NSAID: non-steroidal anti-inflammatory drug; DMARD: disease-modifying anti-inflammatory drug; ^†^ >2 SD above the mean of never-smoking healthy controls without periodontitis, *n* = 50.

**Table 2:** Periodontal characteristics of patients with rheumatoid arthritis (RA) and non-RA controls (NC).

	RA	RA-nonPD	RA-PD	NC	NC-nonPD	NC-PD	*p* Value
N (%)	70 (100)	55 (78.6)	15 (21.4)	70 (100)	53 (75.7)	17 (24.3)	*RA-NC*
Number of teeth (median, IQR)	26 (22–28)	26 (22–28)	26 (22–29)	27 (24–30)	28 (23–31)	27 (25–28)	0.072
PISA in mm^2^ (median, range)	4.9 (0–1319)	0.3 (0–126)	304 (158–1319)	8.0 (0–1897)	0 (0–94)	509 (133–18,970)	0.635
% of sites with pocket probing depth 1–2 mm	96 (74–99)	56 (42–71)	97 (94–99)	93 (75–100)	54 (34–63)	97 (89–100)	
(median, IQR)							
% of sites with pocket probing depth 3–4 mm	4.7 (0.7–24.3)	41 (27–44)	2.3 (0.6–6.1)	5.6 (0–19.4)	37 (22–43)	2.1 (0–8.1)	
(median, IQR)							
% of sites with pocket probing depth ≥5 mm	0 (0–0.8)	3.8 (1.1–13)	0 (0–0)	0 (0–2.6)	6.2 (2.0–23)	0 (0–0)	
(median, IQR)							
% of sites with bleeding on probing	1.1 (0–11)	35 (18–57)	0.6 (0–2.2)	1.1 (0–10)	30 (12–46)	0 (0–2.8)	
(median, IQR)							
Periodontal pathogens detected:							
*P. gingivalis* (Pg) (*n*) (%)	57 (81.4)	45 (81.8)	12 (80.0)	55 (78.6)	43 (81.1)	13 (76.5)	0.834
*A. actinomycetemcomitans* (Aa) (*n*) (%)	11 (15.7)	9 (16.4)	2 (13.3)	21 (30.0)	14 (26.4)	7 (41.2)	0.070
Pg + Aa (*n*) (%)	10 (14.3)	8 (14.5)	2 (13.3)	19 (27.1)	12(22.6)	7 (41.2)	0.094

PD: periodontitis, nonPD: no periodontitis. A PISA of ≥130 mm^2^ has been shown to be associated with the CDC-AAP case definition of periodontitis [24].

**Table 3:** Correlations (Spearman’s ρ) of anti-*P. gingivalis* (Pg) and anti-*A. actinomycetemcomitans* (Aa) antibodies with the subgingival relative abundance of Pg and Aa (number of sequences), periodontal inflamed surface area (PISA), and the RA autoantibodies ACPA and RF.

Correlations	RA (*n* = 70)		NC (*n* = 70)	
**with anti-Pg**	**ρ**	***p*-value**	**ρ**	***p*-value**
Pg relative abundance	0.384	0.001	0.35	0.003
PISA	−0.087	0.472	0.179	0.138
ACPA IgG	0.137	0.259	−0.074	0.542
ACPA IgA	0.207	0.086	−0.054	0.659
RF IgM	0.132	0.276	−0.048	0.691
RF IgA	0.11	0.227	0.058	0.632
**with anti-Aa**	**ρ**	***p*-value**	**ρ**	***p*-value**
Aa relative abundance	0.292	0.014	0.524	<0.000
PISA	−0.117	0.335	0.117	0.333
ACPA IgG	0.011	0.929	0.271	0.024
ACPA IgA	−0.111	0.36	0.111	0.362
RF IgM	0.028	0.816	0.043	0.729
RF IgA	0.056	0.647	0.056	0.645

## Data Availability

Data are available through the second author of the study.
